# Treatment of older adults with *FLT3*-mutated AML: Emerging paradigms and the role of frontline FLT3 inhibitors

**DOI:** 10.1038/s41408-023-00911-w

**Published:** 2023-09-11

**Authors:** Nicholas J. Short, Daniel Nguyen, Farhad Ravandi

**Affiliations:** Department of Leukemia, The University of Texas MD Anderson Cancer Center, Houston, TX USA

**Keywords:** Acute myeloid leukaemia, Acute myeloid leukaemia

## Abstract

*FLT3* is the most frequently mutated gene in acute myeloid leukemia (AML), with *FLT3* internal tandem duplication (ITD) mutations being associated with a more aggressive clinical course. While two large, randomized clinical trials have shown a survival benefit with the frontline use of an oral FLT3 inhibitor (midostaurin or quizartinib) in patients with *FLT3*-mutated AML, the role of FLT3 inhibitors in older adults with newly diagnosed *FLT3*-mutated AML remains unclear. A definitive improvement in survival has not been observed in intensively treated patients over 60 years of age receiving frontline FLT3 inhibitors. Furthermore, many patients with *FLT3*-mutated AML are unsuitable for intensive chemotherapy due to age and/or comorbidities, and this population represents a particular unmet need. For these older patients who are unfit for intensive approaches, azacitidine + venetoclax is a new standard of care and is used by many clinicians irrespective of *FLT3* mutation status. However, *FLT3*-ITD mutations confer resistance to venetoclax and are a well-established mechanism of relapse to lower-intensity venetoclax-based regimens, leading to short durations of remission and poor survival. Preclinical and clinical data suggest synergy between FLT3 inhibitors and venetoclax, providing rationale for their combination. Novel strategies to safely incorporate FLT3 inhibitors into the standard hypomethylating agent + venetoclax backbone are now being explored in this older, less fit population with newly diagnosed *FLT3*-mutated AML, with encouraging early results. Herein, we discuss the frontline use of FLT3 inhibitors in older adults with *FLT3*-mutated AML, including the potential role of FLT3 inhibitors in combination with intensive chemotherapy and as part of novel, lower-intensity doublet and triplet regimens in this older population.

## Introduction

Since 2017, there has been a remarkable expansion of new drug approvals for the treatment of acute myeloid leukemia (AML), both in the frontline and relapsed or refractory settings [1]. These consist of novel chemotherapeutics that are largely agnostic to specific molecular mutations, as well as genomically-targeted therapies, including small molecular inhibitors of FLT3, IDH1, and IDH2 [2]. In particular, great progress has been made in the treatment of older adults with AML who are unfit for intensive chemotherapy. These patients account for a substantial proportion of new AML cases and represent a unique clinical challenge due their poorer tolerance of conventional chemotherapy and a higher incidence of adverse-risk cytomolecular features, as compared with their younger counterparts [3–5]. Fortunately, outcomes for this older population have substantially improved with the clinical development of the oral Bcl-2 inhibitor venetoclax [1, 6–10]. Compared with azacitidine alone, the combination of azacitidine and venetoclax significantly increased response rates and overall survival (OS) in older adults with newly diagnosed AML, and represents the new standard of care for this population [11]. However, fms-like tyrosine kinase 3 (*FLT3*) mutations—particularly *FLT3*-internal tandem duplication (ITD)—are a major mechanism of resistance to venetoclax-based therapies [12, 13], and novel strategies to overcome this *FLT3*-mediated resistance are needed. While intensive chemotherapy plus a FLT3 inhibitor is now standard of care for younger, fit patients with newly diagnosed *FLT3*-mutated AML [14, 15], the role of frontline FLT3 inhibitors in older, unfit patients is not well-established nor is the optimal way to integrate these agents into lower-intensity frontline regimens. In this review, we discuss the impact of *FLT3* mutations in older adults with AML and the historical outcomes of *FLT3*-mutated AML in this population. We review the role of upfront FLT3 inhibitors and specifically highlight the ongoing efforts to develop more effective frontline therapies for older adults with *FLT3*-mutated AML who are unfit for intensive chemotherapy, including novel triplet regimens that integrate FLT3 inhibitors into lower-intensity backbones.

## Incidence of FLT3 mutations

**Fig. 1:**
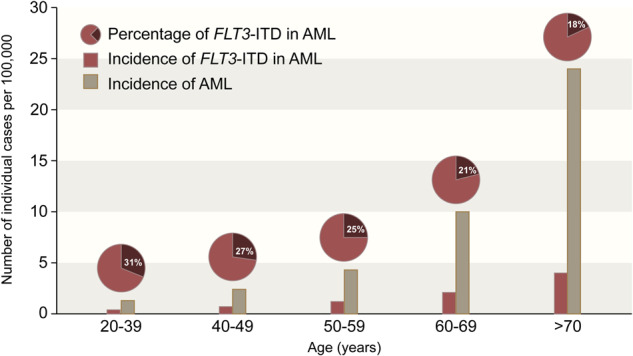
Incidence of *FLT3*-ITD-mutated AML by age. While the proportion of AML cases harboring a *FLT3*-ITD mutation decreases with age, the absolute incidence of *FLT3*-ITD-mutated AML increases with age, driven by overall higher incidences of new AML cases in older patients. Data adapted from Nagel et al. 2017, Schneider et al. 2012, and SEER database [23–25].

*FLT3* mutations are identified in approximately one-third of patients with newly diagnosed AML, are enriched in AML with a normal karyotype, and are frequently co-mutated with *NPM1* and/or *DNMT3A* mutations [16–20]. *FLT3*-ITD mutations are identified in 20–25% of patients with newly diagnosed AML, whereas point mutations in the tyrosine kinase domain (TKD) are identified in 5–10%, approximately half of which occur at D835 in the activation loop [21, 22]. However, the incidence of *FLT3* mutations varies across age groups, and they are relatively less common in older patients in AML [23]. In one study of 3525 adults with newly diagnosed AML, the incidence of *FLT3*-ITD mutations ranged from 23% in patients <45 years of age to 15% in those >70 years of age. Similarly, the incidence of *FLT3*-TKD mutations ranged from 9% to 4% across these age groups, respectively [24]. However, AML is largely a disease of older age with a median age at diagnosis of 69 years [25]. Thus, while *FLT3* mutations are less common in older adults in *relative* terms, due to the higher incidence of AML in older patients, the population-based *absolute* incidence of *FLT3*-mutated AML actually increases with age, with patients >60 years of age representing approximately 60% of all patients with *FLT3*-ITD mutated AML **(**Fig. 1**)** [26]. These figures help to highlight the clinical burden of *FLT3*-mutated AML in this older population and the unmet clinical need that it represents.

## Prognostic impact of FLT3 mutations in older adults with AML

*FLT3-ITD* mutations have been consistently shown across most studies to be associated with a more aggressive phenotype and worse survival in AML, while most studies have shown no—or only minimal—prognostic impact of *FLT3*-TKD mutations [19, 20, 27–30]. In patients with *FLT3*-ITD-mutated AML, a higher allelic ratio is associated with inferior outcomes, particularly when frontline FLT3 inhibitors are not used [31]. Most of these studies have been evaluated in younger adults with AML treated intensively, and there are relatively few studies evaluating the impact of *FLT3* mutations in older patients. In this older population, the prognostic impact of *FLT3* mutations has been less consistent. In one analysis of 243 patients >60 years of age with newly diagnosed AML with normal karyotype treated with intensive chemotherapy, the presence of a *FLT3*-ITD mutation was associated with shorter disease-free survival (DFS) (hazard ratio [HR] 2.1 [95% CI, 1.36–2.23]; *P* < 0.001) and OS (HR 1.97 [95% CI, 1.45–2.68]; *P* < 0.001) in patients 60–69 years of age, but not in those patients who were >70 years of age [32]. In a larger study of patients >60 years of age treated with conventional 7 + 3 induction, *FLT3*-ITD mutations were independently associated with worse OS in a multivariate analysis, with high *FLT3-*ITD allelic ratios associated with a higher risk of death than were lower allelic ratios (HR 1.85 [95% CI, 1.31–2.62]; *P* = 0.0005 and HR 3.51 [95% CI, 2.03–6.08]; *P* < 0.0001, respectively) [33]. However, several other studies failed to show a negative impact of *FLT3*-ITD mutations in intensively treated older adults with newly diagnosed AML [28, 34, 35]. The reasons for the discrepant findings in these studies of intensively treated older adults is unclear but may be due at least in part to smaller patient numbers and underpowered analyses in some of the negative studies.

Studies of *FLT3* mutations in older patients treated with lower-intensity therapies are even more limited. A subgroup analysis of the AZA-AML-001 study—which randomized patients >65 years of age with newly diagnosed AML to azacitidine versus conventional care regimens (CCR)—found no difference in outcomes of *FLT3*-mutated versus *FLT3* wild type patients, although only 18 patients were included in this analysis, limiting its power [36]. In a pooled analysis of patients treated in the VIALE-A trial and the preceding phase 1b study of azacitidine + venetoclax, there was no clear difference in outcomes of patients with *FLT3*-mutated AML (including both ITD and TKD) versus *FLT3* wild type AML when treated with the venetoclax-based combination [13]. However, when further stratified by type of *FLT3* mutation, those with *FLT3*-ITD-mutated AML had particularly poor outcomes (median OS 9.9 months), which was shorter than the *FLT3* wild type population (median OS 14.7 months) and the *FLT3*-TKD-mutated population (median OS 19.2 months). Patients who were *FLT3*-ITD-mutated but *NPM1* wild type AML had the shortest duration of remission among the subgroups evaluated (median 10.1 months versus 18.4 months for *FLT3* wild type AML). Together, these data suggest that *FLT3*-ITD mutations may be a poor prognostic factor even when azacitidine + venetoclax is given.

## Frontline intensive chemotherapy + FLT3 inhibitor in *FLT3-m*utated AML

### Outcomes in younger adults

The combination of a FLT3 inhibitor with frontline intensive chemotherapy is the standard of care for fit adults with newly diagnosed *FLT3*-mutated AML, based on positive data from the RATIFY study that evaluated midostaurin—and, more recently, from the QuANTUM-First study evaluating quizartinib [14, 37]. The RATIFY study was a global, randomized phase III study that randomized 717 adults <60 years of age with newly diagnosed *FLT3*-mutated AML (either ITD or TKD) to standard chemotherapy (i.e. induction with “7 + 3” chemotherapy and high-dose cytarabine consolidation) plus either midostaurin (50 mg orally twice daily) or placebo. Patients also received 1-year of maintenance with midostaurin or placebo. Midostaurin did not significantly improve the complete remission (CR) rate (59% versus 54% in the placebo group; *P* = 0.15) but was associated with a significant improvement in median OS (74.7 months versus 25.6 months; HR for risk of death 0.78 [95% CI, 0.63-0.96]; *P* = 0.009). The survival benefit of midostaurin was seen in both *FLT3-*ITD and *FLT3*-TKD mutations, including in patients with either high or low *FLT3*-ITD allelic ratios. The greatest benefit to midostaurin therapy was observed in patients who underwent allogeneic hematopoietic stem cell transplantation (HSCT) in first remission [15]. These data provided the first definitive evidence for the benefit of frontline FLT3 inhibitors in *FLT3*-mutated AML and led to the Food and Drug Administration (FDA) approval of midostaurin in combination with standard induction and consolidation chemotherapy in adults with newly diagnosed *FLT3*-mutated AML, although the FDA did not approve its use as maintenance therapy. Despite the impressive survival benefit of midostaurin in the RATIFY study, it is important to note that the study did not enroll patients 60 years of age and older, and so the generalizability of these data to this older population is uncertain.

The randomized phase III QuANTUM-First study later evaluated the same backbone chemotherapy regimen in combination with quizartinib (40 mg orally daily) or placebo in adults up to 75 years of age with *FLT3*-ITD-mutated AML who were deemed suitable for intensive chemotherapy [37]. Five-hundred thirty-nine patients were randomized; the median age was 56 years, and 40% of subjects were between 60 and 75 years of age—an older population not included in the RATIFY study. The quizartinib-containing arm had significantly superior survival (median OS: 31.9 months versus 15.1 months in the placebo arm; HR 0.78, [95% CI, 0.62–0.98]; *P* = 0.032), which was driven largely by lower relapse rates (3-year cumulative incidence of relapse 34% [95% CI, 26%–42%] and 45% [95% CI, 37%–53%], respectively). Interestingly, in a *post hoc* analysis, the OS benefit of quizartinib appeared to be limited to patients <60 years of age (median OS not reached versus 23.0 months; HR 0.68 [95% CI, 0.49-0.95]) and was not observed in patients ≥60 years of age (median OS 17.5 months versus 14.2 months; HR 0.91 [95% CI, 0.66–1.26]). In July 2023, quizartinib was approved by the FDA for use in combination with standard induction and consolidation chemotherapy in patients with newly diagnosed *FLT3*-ITD-mutated AML; importantly, it was also approved as monotherapy maintenance for up to 3 years in patients who do not undergo subsequent HSCT. Together, the RATIFY and QuANTUM-First studies provide important clinical data showing that upfront integration of FLT3 inhibitors in patients with newly diagnosed *FLT3*-mutated AML improves outcomes. Specifically, both studies show that the combination of a FLT3 inhibitor with intensive chemotherapy results in improved OS in patients up to 60 years of age, although the data supporting this approach in older adults are still limited.

### Outcomes in older adults

Historically, intensive chemotherapy was the only treatment option for newly diagnosed AML with a reasonable chance of durable remission, and therefore patients up to approximately 75 years of age were routinely recommended to receive intensive AML-directed therapy. However, appropriate selection of patients for intensive chemotherapy is imperative, particularly for patients ≥60 years of age, a group that can have unacceptably high rates of morbidity and mortality with this approach [38].

Patients >60 years of age were excluded from the RATIFY study [14], and therefore randomized data supporting the use of frontline midostaurin in this population is lacking. In a single-arm phase II study from the German-Austrian AML Study Group (AMLSG), adults up to 70 years of age with newly diagnosed *FLT3*-ITD-mutated AML received intensive chemotherapy plus midostaurin, followed by allogeneic HSCT and 1 year of midostaurin maintenance [39]. The findings were then compared to a historical cohort of patients from previous AMLSG trials using propensity score weighting and covariate adjustment. Among the 128 patients 61–70 years of age who were treated, the CR/CRi rate was 72.4% and 38% underwent allogeneic HSCT in first remission. In this older population, the median event-free survival (EFS) was 11.7 months (versus 2.53 months in the control; HR 0.42 [95% CI, 0.29-0.59]; *P* < 0.001) and the median OS was 22.7 months (versus 8.4 months in the control; HR 0.47 [95% CI, 0.33-0.67]; *P* < 0.001), suggesting benefit to adding midostaurin in this group.

As previously described, while older patients who were deemed suitable for intensive chemotherapy were included in the QuANTUM-First study, a survival benefit was not observed in the subgroup of patients. While the precise reasons for the lack of benefit in the older population is not entirely clear, the quizartinib arm had a higher rate of death in the first 3 months of therapy in this group, which may have at least in part offset a potential survival benefit. Until further data are available, these findings provide some skepticism for the use of upfront intensive chemotherapy plus a FLT3 inhibitor in older adults with *FLT3*-mutated AML. Data from these intensive chemotherapy-based prospective studies in older adults, as well as data from prospective studies of lower-intensity regimens are summarized in Table 1.

**Table 1:** Data from frontline, prospective studies on *FLT3*-mutated AML in older adults.

Study	*FLT3* mutation status	Treatment regimen	N	Clinical activity	Study notes
**Intensive Chemotherapy** + **FLT3 inhibitor**					
Sierra J et al. 2020 [61]	ITD and TKD	7 + 3 plus midostaurin or 5 + 2 plus midostuarin	142	CR/CRi: 78%	• Subgroup analysis of age >60 cohort
Dohner H et al. 2022 [39]	ITD only	7 + 3 plus midostaurin	128	CR/CRi: 72% EFS: 11.7 months Median OS: 22.7 months	• Subgroup analysis of age 61–70 cohort
Erba H et al. 2023 [37]	ITD only	7 + 3 plus quizartinib vs 7 + 3 plus placebo	216	Quizartinib (*n* = 107): Median OS 17.5 months Placebo (*n* = 109): Median OS 14.2 months	• Subgroup analysis of age >60 cohort
**Lower-Intensity Doublet with FLT3 Inhibitor**					
Swaminathan M et al. 2021 [42]	ITD only	Azacitidine + quizartinib or LDAC + quizartinib	34	CRc: 79% Median RFS: 8.0 months Median OS: 12.4 months	• Decision to use azacitidine or LDAC based on clinician’s choice
Dennis M et al. 2021 [43]	ITD only	LDAC alone vs LDAC + quizartinib	27	LDAC alone (*n* = 14): CRc: 0% Median OS: 4.2 months LDAC/Quiz (*n* = 13): CRc: 38% Median OS: 13.7 months	• Enrolled patients irrespective of FLT3 status; data are subgroup analysis of FLT3-ITD cohort
Ohanian M et al. 2018 [41]	ITD only	Azacitidine + sorafenib	27	ORR: 78% Median DoR: 14.5 months Median OS: 8.3 months	
Wang ES et al. 2022 [45]	ITD (*n* = 98), TKD (*n* = 21), Co-mutation (*n* = 4)	Azacitidine alone vs Azacitidine + gilteritinib	123	Aza alone (*n* = 49): CRc: 26.5% Median OS: 8.87 months Aza/Gilt (*n* = 74): CRc: 58.1% Median OS: 9.82 months	
**Lower-Intensity Triplet with FLT3 Inhibitor**					
Maiti A et al. 2021 [56]	ITD (*n* = 8), TKD (*n* = 3), Co-mutation (*n* = 1)	Decitabine + venetoclax + FLT3i of choice [gilteritinib, n = 5 sorafenib, n = 5 midostaurin, n = 2]	12	CRc: 92% Median OS: Not reached 2-year OS: 80%	• Decision for FLT3 inhibitor per clinician’s choice
Yilmaz M et al. 2022 [57]	ITD only	Decitabine + venetoclax + quizartinib	6	CR/CRi: 100% Median OS: 14.5 months	
Short NJ et al. 2022 [58]	ITD (*n* = 22), TKD (*n* = 8)	Azacitidine + venetoclax + gilteritinib	30	CR/CRi: 100%; CR: 90% Median OS: Not reached 2-year OS: 71%	

*ITD* internal tandem duplication, *TKD* tyrosine kinase domain, *LDAC* low-dose cytarabine, *CR* complete remission, *CRi* complete remission with incomplete hematologic recovery, *CRc* composite CR rate, *EFS* event-free survival, *DoR* duration of response, *RFS* relapse-free survival, *OS* overall survival.

## Lower-Intensity Doublets with FLT3 Inhibitors in *FLT3-*Mutated AML

As many older adults are not suitable candidates for intensive chemotherapy, several clinical trials have investigated lower-intensity therapies incorporating a FLT3 inhibitor in this population. Based on encouraging data from a phase I/II study of azacitidine + sorafenib in relapsed/refractory *FLT3*-mutated AML [40], this combination was then evaluated as frontline therapy for patients >60 years of age with *FLT3*-mutated AML [41]. Twenty-seven patients were treated with a median age of 74 years (range, 61–86 years). The overall response rate was 78% (including 26% with CR and 45% with CR with incomplete platelet recovery [CRp]). The median duration of response was 14.5 months, and median OS was 8.3 months.

Another study evaluated azacitidine or low-dose cytarabine (LDAC) in combination with quizartinib in patients with *FLT3*-ITD-mutated AML, including patients with relapsed/refractory disease or newly diagnosed patients who were >60 years of age and/or considered to be unfit for intensive chemotherapy [42]. Thirty-four newly diagnosed patients were treated, with a median age of 65 years (range, 58–82 years); 15 patients received azacitidine + quizartinib and 19 received LDAC + quizartinib. The composite CR (CRc) rate was 79%, and the median relapse-free survival (RFS) and OS were 8.0 months and 12.4 months, respectively. Together with the study of azacitidine + sorafenib, these 2 studies show that very high rates of response can be achieved with lower-intensity FLT3 inhibitor doublets in newly diagnosed *FLT3*-mutated AML, although survival remains modest with these approaches.

Building upon the encouraging data with a quizartinib-based doublet, a subsequent study randomized older adults with newly diagnosed AML (irrespective of *FLT3* mutation status) to LDAC with or without quizartinib [43]. A total of 209 patients were randomized (median age 77 years), including 27 patients (16%) with a *FLT3*-ITD mutation and 6 patients (3%) with a *FLT3*-TKD mutation. In the global population there was no difference in response rates or survival outcomes. However, in a subgroup analysis of the 27 patients with a *FLT3*-ITD mutation, the quizartinib-containing arm was associated with higher rates of CR/CRi (38% versus 0%; *P* = 0.05) and superior OS (median OS 13.7 months versus 4.2 months; *P* = 0.04). While the number of patients in this subgroup analysis are relatively small, the findings are suggestive of a benefit to the addition of a FLT3 inhibitor to a lower-intensity backbone in older adults with newly diagnosed *FLT3*-mutated AML who are unfit for intensive chemotherapy.

Gilteritinib is a potent FLT3 inhibitor that is FDA-approved for the treatment of relapsed/refractory *FLT3*-mutated AML based on increased response rates and improved survival compared with chemotherapy alone observed in the randomized phase III ADMIRAL study [44]. To evaluate the possible benefit of gilteritinib in the frontline setting, the LACEWING study randomized patients >65 years of age who were unsuitable for intensive chemotherapy in a 2:1 allocation to either azacitidine + gilteritinib or azacitidine alone [45]. One hundred twenty-three patients were randomized (74 to azacitidine plus gilteritinib and 49 to azacitidine), and the median ages in each cohort were 78 years (range, 59–90 years) and 76 years (range, 61–88 years), respectively. The majority of patients in each group had a *FLT3*-ITD mutation, with or without a concomitant TKD mutation (81% and 86%, respectively). The azacitidine + gilteritinib doublet was associated with a significantly higher rate of CRc (58.1% versus 26.5%; *P* < 0.001), with an improvement in CRc rates that was particularly notable in patients with a *FLT3*-ITD allelic ratio >0.5 (71.4% versus 20.8%; *P* = 0.003). However, despite the increase in CRc rates with the doublet, there was no difference in OS (the primary endpoint of the study) between the 2 arms. The median OS was 9.8 and 8.9 months, respectively (HR 0.92 [95% CI, 0.53–1.56]; *P* = 0.75), and the study was terminated early due to futility. The lack of survival benefit may be explained—at least in part—by different rates of subsequent AML-directed treatment after discontinuing protocol therapy. Fifteen patients (20.3%) in the azacitidine + gilteritinib arm received subsequent therapy, 3 of whom received a subsequent FLT3 inhibitor, compared with 22 patients (44.9%) in the azacitidine arm, 14 of whom received a FLT3 inhibitor. However, it is notable that EFS was also not significantly different between the 2 arms, and therefore the lack of survival benefit cannot be explained entirely by differences in post-protocol therapy. Ultimately, the LACEWING study failed to show a significant benefit to the addition of gilteritinib to azacitidine in older patients with newly diagnosed *FLT3-*mutated AML and suggests that use of these doublets may not be optimal in this population.

## Lower-intensity triplets with FLT3 inhibitors in *FLT3-*mutated AML

### Outcomes of *FLT3*-mutated AML with hypomethylating agent (HMA)/LDAC + venetoclax

The outcomes of older adults with AML have greatly improved with the clinical development of venetoclax-based lower-intensity therapies. In the randomized phase III VIALE-A study, patients ≥75 years of age or unsuitable for intensive chemotherapy with newly diagnosed AML who received azacitidine + venetoclax had improved response rates (CRc rate: 66.4% versus 28.3%; *P* < 0.001) and survival (median OS: 14.7 months versus 9.6 months; *P* < 0.001), as compared with azacitidine alone [11]. In a similar older population, the combination of LDAC + venetoclax (as compared with LDAC alone) also significantly improved response rates and OS [46]. These regimens now represent new standards of care in this older, unfit population with newly diagnosed AML. However, despite the substantial progress that these new venetoclax-based therapies represent, the outcomes of patients with *FLT3*-mutated AML—as well as some other high-risk subgroups—remain suboptimal. For example, in a study of 81 patients who received either azacitidine or LDAC in combination with venetoclax, expansion of pre-existing *FLT3* clones or development of new *FLT3* mutations were commonly seen at the time of relapse [12]. In vitro studies have shown that *FLT3*-ITD mutations lead to overexpression of BCL-xL and MCL-1, both anti-apoptotic proteins shown to contribute to venetoclax resistance [47–53]. While response rates with HMA/LDAC + venetoclax are similar in patients with *FLT3*-mutated and *FLT3* wild type AML, the clonal expansion of *FLT3-*mutated cells in response to these therapies suggest that these mutations are an important mechanism of relapse.

Given the survival benefit observed with azacitidine + venetoclax, many clinicians offer this regimen to older, unfit adults with newly diagnosed AML, regardless of *FLT3* mutation status. A pooled analysis of patients with *FLT3*-mutated AML treated on prospective clinical trials with azacitidine + venetoclax or azacitidine alone was conducted to clarify the clinical outcomes in this population with these commonly used lower-intensity regimens [13]. In this analysis, 42 patients with *FLT3*-mutated AML received azacitidine + venetoclax, and 22 patients had received azacitidine alone (all of whom were treated on the control arm of the VIALE-A study). Across both groups, 41 patients had a *FLT3*-ITD mutation alone, 21 had a *FLT3*-TKD mutation alone, and 2 patients had both. This subgroup analysis confirmed that azacitidine + venetoclax improves response rates in patients with *FLT3*-mutated AML (CRc rates: 67% versus 36% with azacitidine alone). The combination regimen was also associated with numerically superior OS (median OS: 12.5 months versus 8.6 months with azacitidine alone). Among patients treated with azacitidine + venetoclax, there was no difference in OS between those *FLT3*-mutated or *FLT3* wild type AML. However, there was evidence of differential outcomes between the different types of *FLT3* mutations, which may have confounded this analysis. The CRc rates for patients with a *FLT3*-ITD or *FLT3*-TKD mutation were 63% and 77%, respectively. The median OS in patients with *FLT3*-TKD-mutated AML was 19.2 months, compared with only 9.9 months in those with *FLT3*-ITD-mutated AML. This relatively poor survival highlights that effective treatment of *FLT3*-ITD-mutated AML represents a particular unmet need even in the era of venetoclax-based lower-intensity therapies.

### Preclinical and clinical rationales for venetoclax + FLT3 inhibitor combinations

**Fig. 2:**
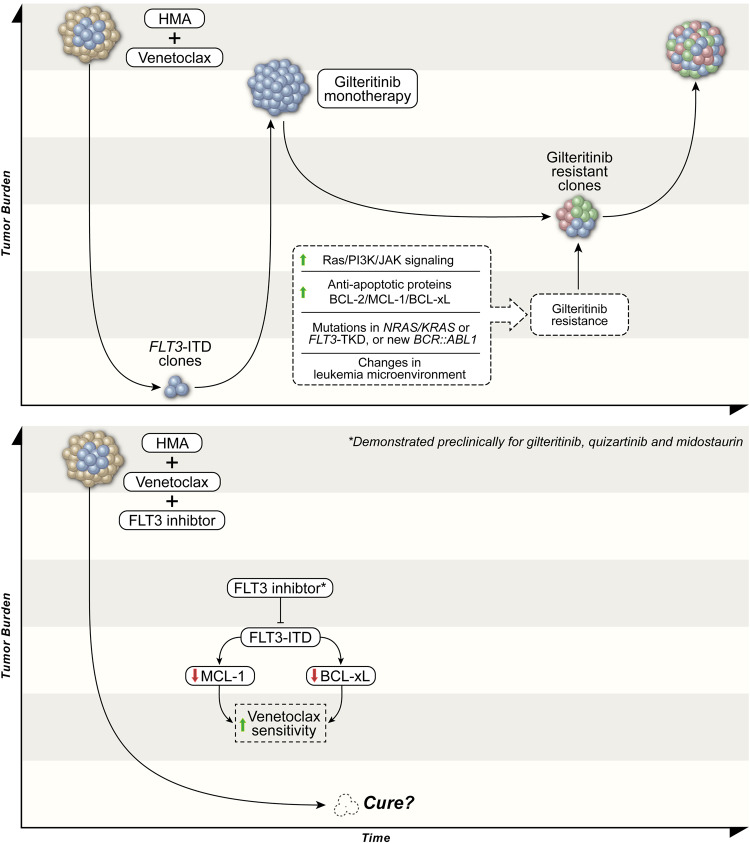
Theoretical disease trajectories of *FLT3*-mutated AML with different treatment approaches. When treated with a doublet regimen of HMA plus venetoclax (top panel), a population of the *FLT3*-mutated subclone persists and contributes to relapse. Sequential therapy with gilteritinib is non-curative even in responding patients, and eventually resistance mechanisms contribute to relapse. A triplet regimen of HMA plus venetoclax plus a FLT3 inhibitor (bottom panel) has added synergy that may lead to longer, more durable responses, and possibly cure. The addition of a FLT3 inhibitor not only targets the *FLT3*-mutated clone, but some FLT3 inhibitors have been shown to increase the sensitivity of leukemia cells to gilteritinib by promoting a more pro-apoptotic phenotype.

*FLT3-*ITD mutations have been shown preclinically to reduce BCL-2 dependence of AML cells and to lead to upregulation of BCL-xL and MCL-1, antiapoptotic proteins that are established to impart resistance to venetoclax [47–53]. Several studies have also shown that various FLT3 inhibitors (including midostaurin, quizartinib, and gilteritinib) increase BCL-2 dependence and reduce expression of BCL-xL and MCL-1, thereby synergistically inducing apoptosis and sensitizing *FLT3*-mutated AML cells to venetoclax [48, 51–53]. Mechanisms of *FLT3*-mediated resistance to venetoclax and synergy between venetoclax and FLT3 inhibitors that may lead to more durable responses in *FLT3*-mutated AML are highlighted in Fig. 2. Interestingly, similar synergy of venetoclax and gilteritinib has also been reported in *FLT3* wild type cell lines [47]. Gilteritinib leads to proteasomal degradation of MCL-1 and increased venetoclax sensitivity, and thus, the combination of venetoclax and gilteritinib may have a possible clinical role even in non-*FLT3*-mutated AML.

These preclinical observations of synergistic activity of venetoclax and FLT3 inhibition were subsequently evaluated in a phase 1b clinical trial of venetoclax + gilteritinib in patients with relapsed/refractory *FLT3*-mutated AML [54]. Among 56 patients with *FLT3*-mutated AML, 47 patients (84%) had a *FLT3*-ITD mutation (either alone or in combination with a TKD mutation). The modified CRc rate (including CR, CRi, CRp, and morphologic leukemia-free state [MLFS]) was 75%, and the median OS was 10.0 months. While it is challenging to compare across clinical trials, the response rates compare favorably to the ADMIRAL trial, where the CRc rate was 54.3%. The higher response rates with venetoclax + gilteritinib were observed despite this study enrolling more heavily pretreated patients than the ADMIRAL study, including 36 patients (64%) with prior FLT3 inhibitor exposure (versus only 13% in ADMIRAL) and 38% of patients having received ≥3 lines of therapy (whereas ADMIRAL only enrolled primary refractory or first salvage patients). This study therefore provides clinical rationale for the further development of venetoclax and FLT3 inhibitor combinations in *FLT3*-mutated AML.

### Clinical experience with HMA plus venetoclax plus FLT3 inhibitor triplets

Building upon the efficacy of the HMA + venetoclax regimen in patients with newly diagnosed AML, several studies are now evaluating lower-intensity therapy + venetoclax + FLT3 inhibitor triplets in patients with newly diagnosed *FLT3*-mutated AML. In a single-center retrospective analysis, 87 patients with newly diagnosed *FLT3*-mutated AML were treated with either triplet therapy (*n* = 27) or doublet therapy with a lower-intensity therapy plus a FLT3 inhibitor, without venetoclax (*n* = 60) [55]. Patients in the triplet cohort were more likely to have received gilteritinib (44%) and those in the doublet group were more likely to have received sorafenib (60%). Triplet therapy resulted in significantly higher rates of CR (67% versus 38%; *P* < 0.01) and CR/CRi (93% versus 70%), which translated to superior OS with the triplets (median OS: not reached versus 9.5 months; *P* < 0.01), a survival benefit that was confirmed on multivariate analysis (HR 3.2 [95% CI, 1.2–9.2]; *P* = 0.02).

Subsequent prospective studies have suggested benefits of these triplet regimens, as compared with historical expectations with lower-intensity therapy plus either venetoclax or a FLT3 inhibitor. In a phase II study of a 10-day regimen of decitabine + venetoclax in older/unfit adults with newly diagnosed AML, a subgroup of patients with *FLT3* mutations were enrolled, for whom the treating physician could add a FLT3 inhibitor [56]. Twelve patients with newly diagnosed *FLT3*-mutated AML were treated (8 with *FLT3*-ITD alone, 3 with *FLT3*-TKD, and 1 with both). The FLT3 inhibitor added was sorafenib in 5 patients, gilteritinib in 5 patients, and midostaurin in 2 patients. The CRc rate (CR + CRp + CRi) was 92%, and the median OS was not reached after a median follow-up of 14.5 months. Early data from an ongoing study of decitabine, venetoclax, and quizartinib have also been reported [57]. Five patients with newly diagnosed *FLT3*-ITD-mutated AML have been treated, all of whom responded (2 CR and 3 CRi). In both studies, no early mortality was observed, suggesting that triplet regimens can be delivered safely in this older population.

The triplet combination of azacitidine, venetoclax, and gilteritinib has also been evaluated in older adults and those unfit for intensive chemotherapy with newly diagnosed *FLT3*-mutated AML [58]. This is the largest prospective study to date evaluating a triplet approach as frontline therapy. Thirty patients were treated with a median age of 71 years (range, 18–86 years); 22 patients (73%) had a *FLT3*-ITD mutation, and 8 patients (27%) had a *FLT3*-TKD mutation. A gilteritinib dose of 80 mg daily was used in the combination based on safety established from a preceding phase I study in relapsed/refractory *FLT3*-mutated AML. Increased myelosuppression was observed with the addition of gilteritinib to the azacitidine + venetoclax backbone, and therefore a bone marrow was performed on day 14 of cycle 1, and the gilteritinib and venetoclax were held to allow for count recovery if it showed marrow remission or aplasia. In consolidation, azacitidine was given for 5 days and venetoclax for 7 days to minimize prolonged myelosuppression. With these mitigation strategies in place, the modified CRc rate was 100%, with 27 patients (90%) achieving CR. With a median follow-up of 17 months, the 1-year OS and 2-year OS rates were 84% and 71%, respectively. These results appear to compare favorably to historical expectations with doublet regimens in a similar older population, including with azacitidine + venetoclax, where the CRc rate was 67% and the 2-year OS in patients with *FLT3*-mutated AML was 20–40%. Thus, using the appropriate mitigation strategies to prevent excessive myelosuppression, the triplet regimen of azacitidine, venetoclax, and gilteritinib can be delivered safely and represents a highly effective treatment option for older or unfit adults with newly diagnosed *FLT3*-mutated AML.

## Areas of uncertainty and future directions

The treatment of *FLT3*-mutated AML in older adults who are unfit for intensive chemotherapy represents a particular clinical challenge, given the lack of an approved FLT3 inhibitor for use in this setting. *FLT3* mutations (particularly *FLT3*-ITD mutations) are associated with a more aggressive clinical course and resistance to commonly used regimens in this population, including an HMA + venetoclax. Triplet regimens of an HMA, venetoclax, and a FLT3 inhibitor have yielded promising outcomes; however, many questions still remain. Despite the encouraging response and early survival data with these regimens, the negative results from the randomized LACEWING study temper enthusiasm somewhat [45]. Thus, randomized studies and longer duration of follow-up of these ongoing single-arm studies will be needed to definitively confirm the benefit of this approach. The optimal dosing of these combinations is also uncertain. The addition of a FLT3 inhibitor to an HMA + venetoclax backbone appears to increase myelosuppression, prompting attenuation of the HMA and venetoclax dosing. While the addition of the FLT3 inhibitor may suppress the *FLT3*-containing subclones, it is possible that reducing doses of the other agents could predispose to *FLT3*-negative relapses, which have been reported to account for 40-50% of relapses with frontline regimens incorporating FLT3 inhibitors [59]. A follow-up phase I/II multicenter, randomized dose-ranging and expansion study of azacitidine, venetoclax, and gilteritinib in newly diagnosed *FLT3*-mutated AML will attempt to identify the optimal doses of these agents when given in combination (NCT05520567). The best FLT3 inhibitor use in the frontline setting is also not clear, and randomized studies comparing intensive chemotherapy plus midostaurin to either gilteritinib (NCT04027309) or crenolanib (NCT03258931) in younger, fit patients with newly diagnosed *FLT3*-mutated AML are ongoing. The findings from these studies could also inform future approaches in older and less fit patients.

Finally, the added benefit of FLT3 inhibition in patients with very low-burden *FLT3* mutations and/or those with *FLT3*-TKD-mutated AML is not well-established in older patients. The eligibility cutoffs in the RATIFY and QuANTUM-First studies were a *FLT3* allelic ratio ≥0.03 and a *FLT3* variant allelic frequency ≥0.05, respectively, and thus randomized data supporting the use of FLT3 inhibitors in the context of very low-burden *FLT3*-mutated AML are lacking. In one retrospective analysis of patients (all ages) with newly diagnosed *FLT3*-mutated AML (median allelic frequency 0.03 [range, 0.01-0.09]), the best outcomes were observed in those who received a frontline FLT3 inhibitor and underwent subsequent HSCT, suggesting that this is an effective approach for fit patients with low-burden *FLT3* mutations [60]. How these data might translate to older patients with low-burden *FLT3* mutations who are not candidates for HSCT remains uncertain. For *FLT3*-TKD-mutated AML in younger, fit adults, the RATIFY study showed that this group benefitted from the addition of midostaurin; however, it is unclear whether this applies in the context of lower-intensity venetoclax-based regimens in older adults. While *FLT3*-ITD mutations are clearly important mechanisms of resistance to an HMA + venetoclax, these regimens result in relatively favorable outcomes in patients with *FLT3*-TKD-mutated AML. Thus, it remains unclear whether the potential added toxicity of a FLT3 inhibitor and the need to reduce doses of the HMA and venetoclax in order to deliver the triplet combination will ultimately improve outcomes for these patients, as compared with use of azacitidine + venetoclax alone.
